# Recognizing Gastric Oxyntic Gland Neoplasms: Characteristic Endoscopic Appearance for Improved Detection

**DOI:** 10.1055/a-2889-6638

**Published:** 2026-06-23

**Authors:** Li Chen, Xue-Guo Sun, Fu-Guo Liu, Xiang-Yan Zhang, Jing Zhao, Ti-Dong Shan

**Affiliations:** 1 Department of Gastroenterology 235960 The Affiliated Hospital of Qingdao UniversityQingdaoShandongChina; 2 Department of Pathology 235960 The Affiliated Hospital of Qingdao UniversityQingdaoShandongChina

**Keywords:** endoscopic ultrasonography, gastric cancer, endoscopy upper GI tract, diagnosis and imaging (inc chromoendoscopy, NBI, iSCAN, FICE, CLE), precancerous conditions and cancerous lesions (displasia and cancer), stomach

## Abstract

**Background** Gastric oxyntic gland neoplasms (GOGNs)—including oxyntic gland adenoma (OGA), gastric adenocarcinoma of fundic gland type (GA-FG), and gastric adenocarcinoma of fundic gland mucosa type (GA-FGM)—are rare epithelial neoplasms that often lack overt malignant appearance on endoscopy. This study aimed to characterize the endoscopic features of GOGNs that may facilitate their recognition.

**Methods** We retrospectively analyzed consecutive, histologically confirmed GOGNs diagnosed at a tertiary center between January 2019 and July 2025. Endoscopic features on white-light endoscopy (WLE), magnifying narrow-band imaging (NBI), and endoscopic ultrasonography (EUS) were evaluated.

**Results** Among 156,240 upper endoscopic examinations, 35 patients were diagnosed with GOGNs (0.028%). Lesions were mostly small (median 6 mm) and located in the fundus/upper body (52%), predominantly Paris type 0-IIa (57%) or subepithelial-like elevations (60%). Color was similar to the surrounding mucosa (49%) or mildly reddish (37%), occasionally showing branching vessels (31%) or focal pigmentation (14%). On NBI, OGA and GA-FG lesions ( *n* = 12) frequently showed regularly enlarged crypt openings (92%) and branching vessels (83%) with ill-defined margins (75%), whereas GA-FGM ( *n* = 4) more often showed a distinct demarcation line (75%) with irregular microvascular/microsurface patterns (50%). EUS demonstrated localization mainly within the middle-to-deep mucosal layer and occasional submucosal extension, with intact muscularis propria.

**Conclusions** GOGNs typically present as small, inconspicuous elevated lesions in the fundus or upper stomach, often with near-normal or slightly reddish mucosal color and characteristic surface vascular and magnifying features. Awareness of these endoscopic patterns may improve recognition of GOGNs during routine endoscopy. Larger studies are needed to validate these findings.

## Introduction

Gastric oxyntic gland neoplasms (GOGNs) are a distinct category of gastric epithelial tumors showing differentiation toward fundic gland cell lineages, comprising oxyntic gland adenoma (OGA), gastric adenocarcinoma of fundic gland type (GA-FG), and gastric adenocarcinoma of fundic gland mucosa type (GA-FGM). First described by Tsukamoto in 2007 1 and defined as a novel gastric cancer subtype by Ueyama et al. in 2010, 2 they are uncommon but require accurate endoscopic recognition. According to the 2019 WHO classification, 3 mucosa-confined lesions are classified as OGA, whereas those invading the submucosa are classified as GA-FG. Despite their typically small size and subtle endoscopic appearance, some GOGNs may already show submucosal invasion. 2 4 GA-FGM, a more recently recognized variant, shows both fundic gland and foveolar differentiation and may have endoscopic features distinct from OGA and GA-FG. 5 6 Although many case reports and small case series have described the endoscopic features of GOGNs, their imaging characteristics have not been comprehensively characterized. GOGNs may be under-recognized during routine endoscopy. Their subtle macroscopic appearance—small elevation, near-normal mucosal color, minimal surface irregularity—can mimic benign lesions such as fundic gland polyps (FGPs), potentially resulting in misclassification or incidental removal by cold-snare polypectomy or biopsy without optimal margin control. 7 Enhancing recognition of their characteristic endoscopic patterns may help improve diagnostic accuracy and reduce misclassification.

This retrospective study analyzed a consecutive series of histologically confirmed GOGNs at a single tertiary center, focusing on their white-light endoscopic, magnifying narrow-band imaging (NBI), and endoscopic ultrasonography (EUS) features, with the aim of providing practical visual clues for routine clinical practice.

## Methods

### Study Design and Population

This was a retrospective observational study conducted at a tertiary referral hospital in China. Medical records and endoscopic images obtained between January 1, 2019, and July 1, 2025, were reviewed to identify 35 consecutive patients with histologically confirmed GOGNs. Diagnosis and classification were established by histopathological examination in accordance with the 2019 World Health Organization (WHO) criteria, 3 as described in the *Histopathological diagnosis* section.

### Endoscopic Evaluation

All patients underwent diagnostic upper gastrointestinal endoscopy using high-definition video endoscopes (GIF-HQ290 and GIF-H260Z; Olympus, Tokyo, Japan). EUS was performed using a 20-MHz probe to evaluate the layer of origin and depth of invasion, when indicated. Lesion morphology was assessed according to the Paris classification for superficial neoplastic lesions, 8 9 and the Borrmann system was applied in cases initially suspected of advanced gastric cancer. 10 Magnifying endoscopy with narrow-band imaging (ME-NBI) was performed to observe microsurface and microvascular structures, following the vessel-plus-surface (VS) classification system. 11

*Helicobacter pylori* infection status was determined based on the Kyoto classification 12 in combination with a ^13^ C-urea breath test, and categorized as naïve, current infection, or previous infection. 13 Gastric mucosal atrophy was assessed under white-light endoscopy (WLE) using the Kimura–Takemoto classification, 14 and classified into closed-type or open-type patterns.

### Histopathological Confirmation

Histopathological examination was performed on specimens obtained by endoscopic biopsy, cold-snare polypectomy (CSP), endoscopic submucosal dissection (ESD), or surgical resection. Tumor depth was classified following WHO 2019 guidelines 3 : OGA (mucosal lesions), GA-FG (submucosal invasive lesions), and GA-FGM (mixed fundic gland and foveolar differentiated lesions). 6

Immunohistochemical staining was performed when necessary to support the diagnosis. Background mucosal atrophy and intestinal metaplasia were staged using the OLGA 15 and OLGIM 16 systems.

### Management and Follow-up

Clinical management was determined according to the initial endoscopic impression. Lesions suspected to be GOGNs were generally treated by ESD or surgical resection, whereas lesions not recognized at index endoscopy were sometimes removed incidentally by CSP or forceps biopsy. Follow-up after resection was performed according to relevant guidelines and institutional practice.

### Data Collection

For each case, clinical, endoscopic, and histopathological data were collected, including patient age, sex, *H. pylori* status, background mucosal atrophy, lesion size and location, macroscopic type, conventional endoscopic findings, ME-NBI features, EUS findings, histopathological diagnosis, treatment modality, and follow-up outcomes.

### Ethics Statement

This study was conducted in accordance with the principles of the Declaration of Helsinki (2013 revision) and was approved by the institutional Ethics Committee on July 8, 2025 (Approval No. QYFY-WZLL-30576). Given the retrospective and noninterventional design of the study, the requirement for written informed consent was waived.

## Results

### Clinical Characteristics

During the study period, 35 histologically confirmed cases of GOGNs were identified among 156,240 diagnostic upper gastrointestinal endoscopic examinations, yielding a detection rate of 2.8 per 10,000 (0.028%). Table 1 summarizes clinical characteristics. The mean patient age was 58 years (median 61; range 24–80). The cohort comprised 19 men (54%) and 16 women (46%), with no significant sex difference.

**Table 1:** Clinical characteristics of 35 patients with GOGNs.

Variable	Number of cases	Percentage
**Age (years)**		
Mean age	58	**-**
Median age (range)	61(24-80)	**-**
**Sex**		
Male	19	54%
Female	16	46%
***H. pylori* infection status **		
Negative	32	91%
Current infection	2	6%
Previous infection	1	3%
**Presence of atrophic gastritis**		
Closed-type atrophy	5	14%
Open-type atrophy	3	8%
None	27	78%
**History of PPI use**		
Yes	21	60%
No	14	40%

Abbreviations: GOGNs – gastric oxyntic gland neoplasms, PPI – proton pump inhibitor.

*H. pylori* infection was absent in 32 patients (91%), while 3 patients (9%) had either a current or previous infection. In all, 8 patients (22%) showed endoscopic evidence of global gastric mucosal atrophy, classified as closed-type in 5 cases and open-type in 3 cases according to the Kimura–Takemoto classification. Acid-suppressive medication use was reported in 21 patients (60%).

### Endoscopic Characteristics

**Fig. 1:**
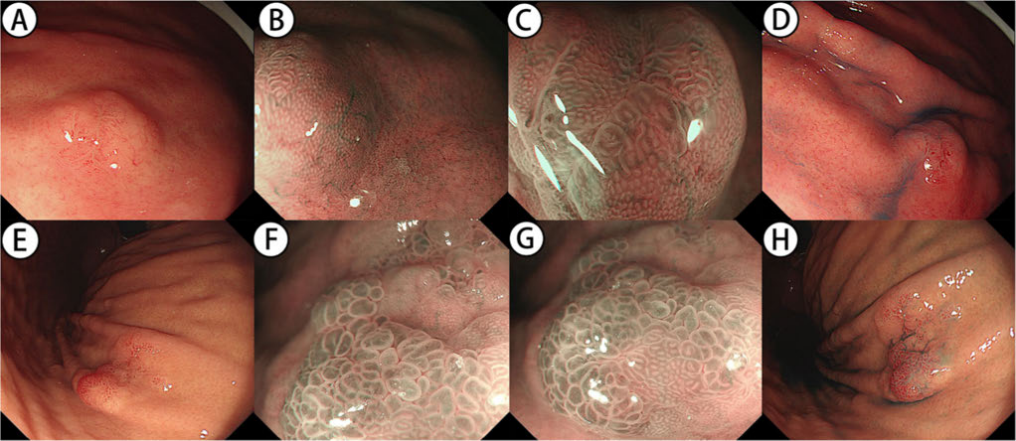
Representative endoscopic findings of typical gastric oxyntic gland neoplasms (GOGNs) located in nonatrophic mucosa. Panels A–D (Case 1): ( **A** ) White-light endoscopy shows an SMT-like, elevated lesion (Paris Type 0–IIa) in the upper third of the gastric body. ( **B** ) NBI reveals no clear demarcation line; enlarged CO, arcuate MCE, and widened IP are present within the elevated area. ( **C** ) NBI magnification highlights enlarged CO and IP, along with greenish submucosal converging veins. ( **D** ) Indigo carmine chromoendoscopy fails to delineate a distinct border. Panels E–H (Case 2): ( **E** ) White-light endoscopy depicts a mildly reddish Type 0–IIa lesion. ( **F** , **G** ) NBI demonstrates relatively uniform villous structures with enlarged CO, arcuate MCE, and widened IP; no clear irregular microsurface or microvascular patterns are present. The surrounding mucosa appears nonatrophic. ( **H** ) Indigo carmine chromoendoscopy again fails to clearly delineate the lesion margin.

**Fig. 2:**
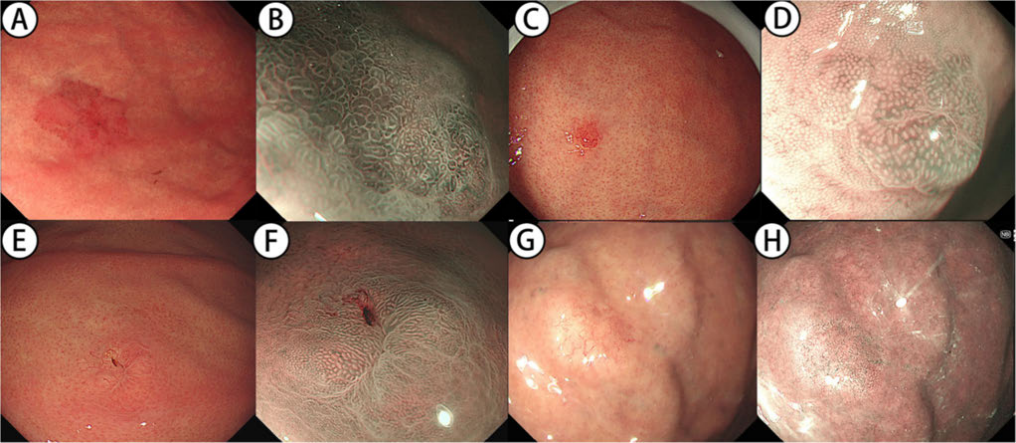
Additional representative endoscopic findings of GOGNs in nonatrophic mucosa. Panels A–B (Case 1): ( **A** ) White-light endoscopy depicts a flat Type 0–IIb lesion in the middle-to-upper gastric body; pigmentation is present within the lesion. ( **B** ) NBI shows enlarged CO accompanied by arcuate MCE and enlarged CO with widened IP. Panels C–D (Case 2): ( **C** ) White-light endoscopy shows a small, reddish Type 0–IIa lesion. ( **D** ) NBI magnification reveals enlarged CO and IP together with greenish submucosal converging veins; surrounding mucosa is nonatrophic. Panels E–F (Case 3): ( **E** ) White-light endoscopy shows a gently sloping elevated lesion with coloration similar to the adjacent mucosa. ( **F** ) NBI demonstrates a relatively uniform microsurface structure with a central biopsy scar. The adjacent mucosa is nonatrophic. Panels G–H (Case 4): ( **G** ) White-light endoscopy shows a slightly yellowish flat Type 0–IIb lesion in the upper gastric body, with branch-like vessels visible on the surface. ( **H** ) NBI fails to reveal a clear demarcation line and shows no definite irregular microsurface or microvascular patterns.

**Fig. 3:**
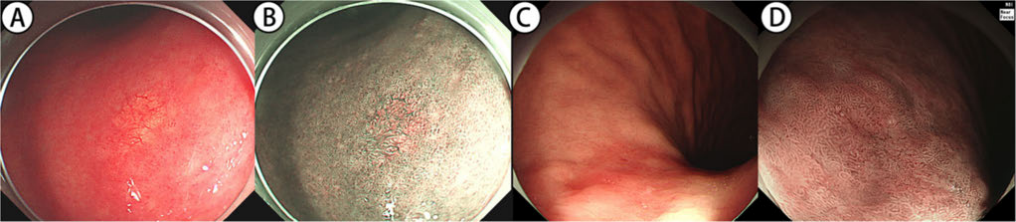
Representative endoscopic findings of GOGNs in atrophic mucosa. Panels A–B (Case 1): ( **A** ) White-light endoscopy shows a slightly yellowish flat Type 0–IIb lesion on the lesser curvature of the middle-to-upper gastric body, with branch-like vessels on the surface. ( **B** ) NBI demonstrates arcuate MCE with regular arrangement and enlarged CO on the lesion surface; surrounding mucosa exhibits atrophic changes. Panels C–D (Case 2): ( **C** ) White-light endoscopy depicts a shallow depressed Type 0–IIc lesion on the posterior wall of the upper gastric body near the cardia, reddish in color. ( **D** ) NBI shows irregular microsurface patterns and irregular, thickened, tortuous microvessels with a clearly demarcated boundary; surrounding mucosa is atrophic.

**Fig. 4:**
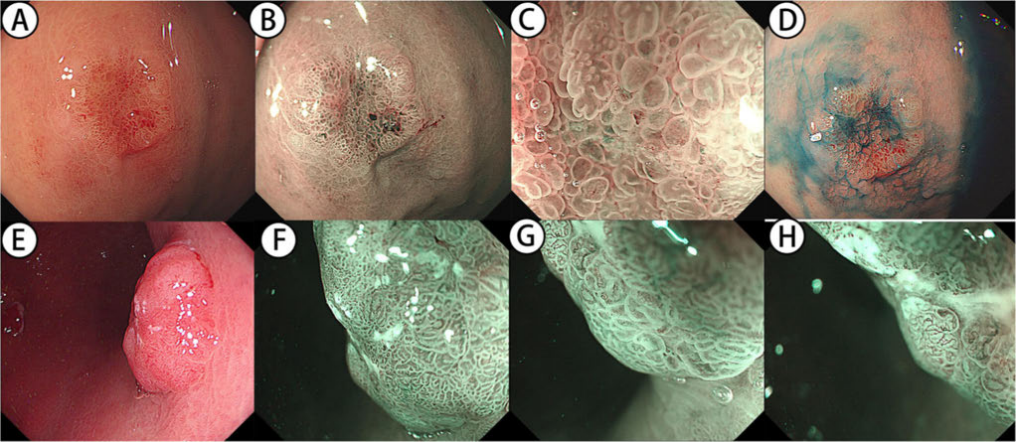
Representative endoscopic findings of gastric adenocarcinoma of GA-FGM. Panels A–D (Case 1): ( **A** ) White-light endoscopy depicts a combined Type 0–IIa+IIc lesion with a mildly reddish surface on a nonatrophic background. ( **B** ) NBI shows dilated CO and IP. ( **C** ) NBI magnification reveals a tumor surface with variably sized granular or leaf-like microsurface structures. ( **D** ) Indigo carmine chromoendoscopy makes the lesion margin more distinct. Panels E–H (Case 2): ( **E** ) White-light endoscopy shows a protruded Type 0–I lesion with a clearly defined border on a nonatrophic background. ( **F** – **H** ) NBI and NBI magnification reveal irregular microsurface patterns, white zones with nonuniform widths, and thickened, tortuous, dilated microvessels.

**Fig. 5:**
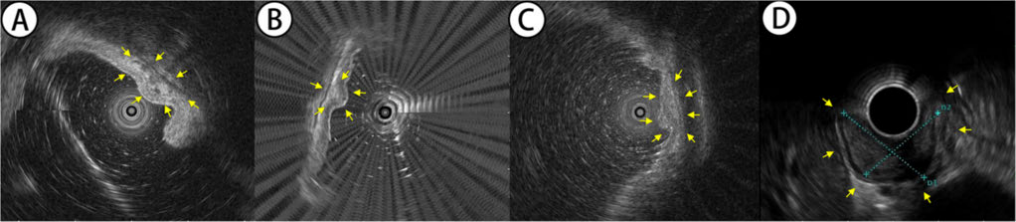
Representative EUS findings of GOGNs. Yellow arrows indicate the lesions in all panels. ( **A** ) Case 1: EUS shows a lesion involving the mucosa and submucosa (layers 2–3) with iso- to hypoechoic thickening; the muscularis propria is intact. Postoperative histology demonstrates fundic-gland-type gastric carcinoma with focal submucosal invasion (450 μm). ( **B** ) Case 2: EUS shows a lesion involving the mucosa and submucosa with iso- to hypoechoic thickening; the muscularis propria remains continuous. ( **C** ) Case 3: EUS shows a lesion within the mucosa and submucosa, hypoechoic in appearance. Postoperative histology demonstrates GA-FG with submucosal invasion (200 μm). ( **D** ) Case 4: EUS shows a lesion involving layers 1–3 with indistinct boundaries between mucosa, muscularis mucosae, and submucosa; the lesion is hypo- to isoechoic with heterogeneous internal echoes. Surgery demonstrates GA-FGM with submucosal invasion.

The endoscopic findings of GOGNs are summarized in Tables 2 and 3 , and representative endoscopic appearances are shown in Figs. 1–5 .

**Table 2:** Endoscopic characteristics of 35 patients with GOGNs.

Variable	Number of cases	Percentage
**Lesion location**		
Fundic dome	4	12%
Cardia	5	14%
Upper third of gastric body	14	40%
Middle third of gastric body	6	17%
Lower third of gastric body	6	17%
**Surrounding mucosa**		
Atrophic	4	11%
Non-atrophic	31	89%
**Lesion size (mm)**		
Mean	8	**–**
Median (range)	6 (2–47)	**–**
**Macroscopic tumor type**		
*Early gastric cancer*		
Type 0-I	2	6%
Type 0-IIa	20	57%
Type 0-IIb	9	25%
Type 0-IIc	2	6%
Type 0-IIa+IIc	1	3%
Type 0-III	1	3%
*Advanced gastric cancer*		
Borrmann type I	0	0
Borrmann type II	0	0
Borrmann type III	0	0
Borrmann type IV	0	0
**Multiple lesions**		
Present	8	23%
Absent	27	77%
**Subepithelial tumor-like appearance (SMT-like)**		
Present	21	60%
Absent	14	40%
**Lesion color**		
Reddish	13	37%
Yellowish	3	8%
Whitish	2	6%
Similar to surrounding mucosa	17	49%
**Branch-like vessels**		
Present	11	31%
Absent	24	69%
**Pigmentation**		
Present	5	14%
Absent	30	86%

Abbreviations: GOGNs – gastric oxyntic gland neoplasms.

**Table 3:** Magnifying NBI and EUS characteristics of GOGNs with definitive histology ( *n* = 16).

Variable	Number of cases	Percentage
**Magnifying NBI findings**		
** OGA and GA-FG ( *n* = 12) **		
Enlarged CO	11	92%
Curved MCE	9	75%
Widened, regular IP	10	83%
Branching dilated surface vessels	10	83%
Ill-defined lesion margins	9	75%
Pronounced irregular MS/MV pattern	0	0
** GA-FGM ( *n* = 4) **		
Enlarged CO	3	75%
Curved MCE	4	100%
Widened, regular IP	4	100%
Branching dilated surface vessels	1	25%
Ill-defined lesion margins	1	25%
Pronounced irregular MS/MV pattern	2	50%
Central depression	1	25%
** EUS findings ( *n* = 16) **		
Lesion location within the mucosal layer		
Superficial–middle	0	0
Middle	2	12.5%
Middle–deep	14	87.5%
Well-defined margins	9	56%
Submucosal extension	13	81%
Muscularis propria intact	16	100%
Echotexture — hypoechoic	4	25%
Echotexture — heterogeneous	12	75%

Abbreviations: NBI – narrow band imaging, EUS – endoscopic ultrasonography; GOGNs – gastric oxyntic gland neoplasms, ESD – endoscopic submucosal dissection, OGA – oxyntic gland adenoma, GA-FG – gastric adenocarcinoma of fundic gland type, GA-FGM – gastric adenocarcinoma of fundic-gland mucosa type, MCE – marginal crypt epithelium, CO – crypt opening, IP – intervening part, MS – microsurface, MV – microvascular pattern.

#### Lesion Distribution and Morphology

Under white-light endoscopy, lesions were predominantly distributed in the upper third of the gastric body (14/35, 40%), followed by the middle third (6/35, 17%), lower third (6/35, 17%), cardia (5/35, 14%), and fundic dome (4/35, 12%). No lesions were detected in the esophagogastric junction or antrum. Solitary lesions were observed in 27 patients (77%), while multiple lesions occurred in 8 patients (23%).

**Fig. 6:**
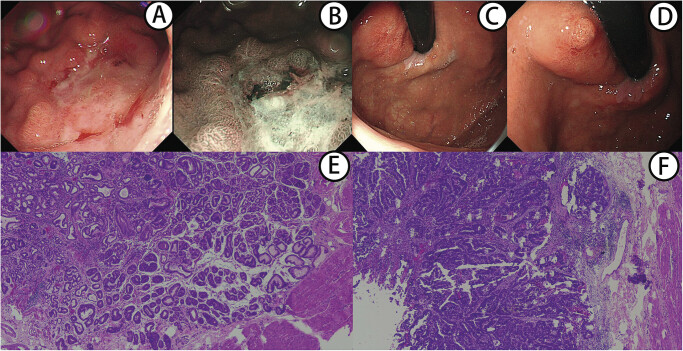
Representative endoscopic findings of two surgically resected cases and histopathological findings of Case 1. Panels A–B (Case 1): ( **A** ) White-light endoscopy shows an ulcerative Type 0–III lesion (47 × 35 mm) in the upper gastric body, with markedly reddish, edematous surrounding mucosa and slight elevation. ( **B** ) NBI shows relatively regular microsurface patterns adjacent to the ulcer, while visualization of the ulcer center is hindered by surface exudate. Panels C–D (Case 2): ( **C** ) White-light endoscopy depicts a protruded Type 0–I lesion (18 × 15 mm) at the cardia, with an SMT-like appearance and a reddish surface, showing widened CO and IP. ( **D** ) Follow-up endoscopy after four months reveals a hemispherical central protrusion; microsurface patterns remain largely unchanged. Panels E–F (Case 1): Histopathology findings of case 1: ( **E** ) Higher-magnification view shows irregularly arranged fundic gland–type glands with mild cytologic atypia and submucosal invasion. ( **F** ) Another area shows more complex fused and cribriform glandular architecture with increased atypia, accompanied by stromal reaction and inflammatory cell infiltration. All pathologic panels: Hematoxylin and Eosin (H&E) staining with hematoxylin counterstaining.

Most lesions were small and superficial, with a median lesion diameter of 6 mm. The lesions demonstrated distinct morphologic patterns according to the Paris endoscopic classification: type 0-IIa morphology was most common (20/35, 57%), followed by type 0-IIb (9/35, 25%), type 0-IIc (2/35, 6%), mixed type 0-IIa+IIc (1/35, 3%), type 0-I (2/35, 6%), and type 0-III (1/35, 3%). Two large lesions, measuring 47 × 35 mm and 18 × 15 mm respectively ( Fig. 6 ), were initially classified as Borrmann type III and type I on white-light endoscopy but were reclassified as type 0-III and type 0-I respectively after histopathology confirmed only submucosal invasion.

Elevated subepithelial tumor-like (SMT) morphology was observed in 21 lesions (60%), while 14 lesions (40%) were nonelevated.

#### Surface Coloration and Vascular Findings

In terms of coloration, 17 lesions (49%) were isocolored with the surrounding mucosa, 13 (37%) were reddish, 3 (9%) yellowish, and 2 (6%) whitish. Branching surface vessels were noted in 11 lesions (31%), and surface pigmentation in five (14%).

#### Perilesional Mucosal Characteristics

Although the majority (31/35, 88%) of GOGNs occurred in nonatrophic mucosa, 4 (12%) lesions were identified within atrophic areas. These lesions appeared flatter or slightly depressed compared with those in nonatrophic mucosa.

#### Magnifying Endoscopy Findings (NBI)

Under magnifying NBI, lesion-specific microstructural patterns were evaluated only in the 16 completely resected lesions with definitive histology (ESD = 14, surgery = 2). Among them, 12 were classified as OGA or GA-FG and 4 as GA-FGM. Typical OGA and GA-FG lesions ( *n* = 12) exhibited branching dilated vessels in 10/12 (83%) and ill-defined lesion margins in 9/12 (75%). Enlarged crypt openings (COs) were present in 11/12 (92%), curved marginal crypt epithelium (MCE) in 9/12(75%), and widened yet regular intervening parts (IP) in 10/12 (83%), with overall microsurface and microvascular structures remaining relatively uniform and orderly. By contrast, among GA-FGM lesions ( *n* = 4), clear demarcation lines were observed in 3 (75%). Irregular microsurface and microvascular patterns were noted in 2 (50%), with coarse or lobulated granular structures and twisted, dilated atypical microvessels.

#### Endoscopic Ultrasonography (EUS) Findings

EUS of the 16 completely resected lesions with definitive histology (all treated by ESD or surgery) showed that most lesions (12/16, 75%) were located in the middle-to-deep mucosal layer, with submucosal extension in 13/16 (81%). Lesions appeared predominantly hypoechoic or heterogeneous in echogenicity; margins were well defined in 9/16 (56%) and indistinct in 7/16 (44%). The muscularis propria was intact in all cases.

### Histopathologic Findings

Histopathologic analysis of these 16 completely resected lesions, classified three (19%) lesions as OGA, nine (56%) as GA-FG, and four (25%) as GA-FGM ( **Supplementary Fig. S1** , **Supplementary Table S1** ). OGA lesions were confined to the mucosa, whereas all GA-FG and GA-FGM lesions invaded the submucosa (50–1000 μm) without muscularis propria or lymphovascular involvement. Immunohistochemical findings were consistent with the histologic subtype, and detailed marker data are provided in **Supplementary Table S2** .

### Management and Follow-up

Sixteen patients underwent definitive treatment, including ESD in 14 and surgical resection in 2 ( **Supplementary Figs. S2 and S3** ; **Supplementary Table S3** , with explanatory details provided in **Supplementary Table S4** ). The remaining 19 patients were managed conservatively because of incidental removal by biopsy or CSP, small residual lesions, or patient-related factors. During a median follow-up of 27 months (range, 13–70), no local recurrence or disease-specific death was observed.

## Discussion

GOGNs, including OGA, GA-FG, and GA-FGM, originate from fundic gland mucosa and most often occur in the upper to middle stomach. They are found predominantly in nonatrophic mucosa, 17 18 yet in our cohort, 4 lesions (11%) occurred within atrophic areas, indicating that residual acid-secreting glands can still serve as the pathologic substrate even in an atrophic background. Clinicians should be aware that small, subtle protrusions in atrophic mucosa can represent GOGNs, warranting careful endoscopic evaluation to avoid missed diagnoses.

In cases where the mucosa immediately surrounding the lesion appeared nonatrophic, 5 patients nevertheless had closed-type gastric atrophy by Kimura–Takemoto classification—three with C-1 type and two with C-2 type. Given the low malignant potential and slow progression of GOGNs, the background mucosa at the time of tumor initiation may have been entirely nonatrophic, with atrophic changes developing later. Recognizing this possible discrepancy between background mucosa at onset and at diagnosis may offer important insights into the pathogenesis and natural history of these tumors.

Although the number of GA-FGM lesions was limited ( *n* = 4), their endoscopic appearance differed somewhat from that of OGA and GA-FG. GA-FGM lesions more often showed clearer borders (3/4, 75%), a mildly reddish and more prominently protruded morphology (3/4, 75%), and, in some cases (2/4, 50%), irregular microsurface and microvascular patterns, occasionally with central depression (1/4, 25%). These findings may reflect superficial exposure of tumor cells with foveolar differentiation, resulting in greater optical contrast and structural irregularity at the mucosal surface. Because only four GA-FGM lesions were available for analysis, these observations should be interpreted cautiously.

Owing to their subtle macroscopic appearance—small, slightly elevated lesions with near-normal mucosal color in some lesions, minimal surface irregularity, and occasionally surface branching vessels—GOGNs can be easily mistaken for fundic gland polyps (FGPs). While FGPs can be safely managed with CSP due to their non-neoplastic nature, 19 GOGNs frequently exhibit submucosal invasion and therefore require en bloc resection with adequate margin assessment rather than CSP. In our cohort of 35 patients, 19 (54.3%) were not recognized as GOGNs at the index endoscopic examination and underwent biopsy or CSP without margin-controlled resection. Notably, coexistence with FGPs was present in 7 of these 19 cases (36.8%), which may have further contributed to their misclassification as benign polyps. Accurate differentiation between GOGNs and FGPs is thus both important and challenging. Takahashi et al. reported that the white ring sign (WRS)—a whitish circumferential halo surrounding the lesion base—is typically seen in FGPs (WRS-positive) but absent in GOGNs (WRS-negative), and can serve as a reliable distinguishing marker. 7 Incorporating WRS assessment into routine endoscopic practice may enhance diagnostic accuracy and reduce the risk of incomplete resection.

However, even with careful observation, distinguishing GOGNs from benign lesions such as FGPs at the index examination can remain challenging. Accurate recognition at this stage is crucial, because misclassification as a benign polyp may result in biopsy or cold snare removal, thereby obscuring the lesion remnant and potentially forfeiting the opportunity for subsequent en bloc resection and optimal pathologic assessment. For indeterminate lesions, diagnostic biopsy may still be reasonable; however, in very small lesions, accurate localization by high-quality endoscopic imaging, clip placement, or submucosal tattooing with India ink 20 or carbon particles 21 should be considered to facilitate subsequent treatment if histopathology confirms GOGN.

This study has several limitations. First, it was a retrospective analysis conducted at a single institution, and the sample size was limited, which may have introduced selection bias and restricted generalizability. Second, detailed ME-NBI and EUS–pathology correlation was available only for the completely resected lesions, and the number of GA-FGM lesions was too small for definitive subtype-specific conclusions. Third, this study evaluated only lesions that were ultimately detected and diagnosed. Accordingly, the endoscopic features of truly missed GOGNs could not be assessed. Therefore, although under-recognition or insufficient awareness may contribute to the low reported detection rate in some settings, it is also possible that missed lesions have less conspicuous or different endoscopic appearances that make them more difficult to detect. Larger multicenter prospective studies are needed to validate these endoscopic findings and to clarify the relationship between lesion subtype, morphology, and invasion depth.

## Conclusion

GOGNs typically arise in the fundus or upper stomach and often present as subtly elevated, type 0-IIa lesions with mucosal color similar to or slightly redder than the surrounding mucosa, branching surface vessels, focal pigmentation and characteristic magnifying features. Recognition of these features may improve endoscopic detection and help avoid misclassification as benign fundic gland lesions. Larger studies are needed to validate these findings.

**Abbreviations**
GOGN(s): Gastric oxyntic gland neoplasm(s)
OGA: Oxyntic gland adenoma
GA-FG: Gastric adenocarcinoma of fundic gland type
GA-FGM: Gastric adenocarcinoma of fundic-gland mucosa type
WHO: World Health Organization
SMT: Submucosal tumor
H. pylori: *Helicobacter pylori*
MCE: Marginal crypt epithelium
CO: Crypt opening
IP: Intervening part
EUS: Endoscopic ultrasonography
NBI: Narrow band imaging
VS classification: Vessel-plus-surface classification for magnifying endoscopy
CSP: Cold-snare polypectomy
ESD: Endoscopic submucosal dissection
FGP: Fundic gland polyp
EMR: Endoscopic mucosal resection
OLGA: Operative Link on Gastritis Assessment staging system
OLGIM: Operative Link on Gastric Intestinal Metaplasia Assessment staging system
CgA: Chromogranin A
MUC: Mucin (glycoprotein family; e.g., MUC2, MUC5AC, MUC6)
NET: Neuroendocrine tumor
WRS: White ring sign
PPI: Proton pump inhibitor
MS: Microsurface
MV: Microvascular

## Data Availability

The datasets generated and analyzed during the current study are not publicly available due to patient confidentiality, but are available from the corresponding author on reasonable request.

## Supplementary Material

Ergänzendes Material Supplementary Material
