# Supplementary material for: Recognizing Gastric Oxyntic Gland Neoplasms: Characteristic Endoscopic Appearance for Improved Detection
Source: Endosc Int Open. 2026 Jun 23;14:a28896638. doi: 10.1055/a-2889-6638 (PMC13289782; doi:10.1055/a-2889-6638)

Supplementary Table S1. Histopathological characteristics of GOGNs resected by ESD or surgery (n=16)

Variable	Cases (n)	Percentage
**Classification**		
OGA	3	19%
GA-FG	9	56%
GA-FGM	4	25%
**Tumor invasion depth**		
pT1a	3	25%
pT1b	13	75%
pT2	0	0
pT3	0	0
pT4	0	0
**Lymph node metastasis**		
Present	0	0
Absent	16	100%
**Vascular invasion**		
Present	0	0
Absent	16	100%

Abbreviations: GOGNs – gastric oxyntic gland neoplasms, ESD – endoscopic submucosal dissection, OGA – oxyntic gland adenoma, GA-FG – gastric adenocarcinoma of fundic gland type, GA-FGM – gastric adenocarcinoma of fundic-gland mucosa type

Supplementary Table S2. Immunohistochemical profiles of GOGNs resected by ESD or surgery (n = 16)

Immunohistochemistry	Cases (n)	Percentage
MUC5AC positive	4	25%
MUC5AC negative	12	75%
MUC6 positive	16	100%
MUC6 negative	0	0
P53 positive	13	81%
P53 negative	3	19%
Ki-67 positive	5	31%
Ki-67 negative	11	69%
Pepsinogen-1 positive	16	100%
Pepsinogen-1 negative	0	0
H^+/K^+-ATPase positive	14	86%
H^+/K^+-ATPase negative	2	14%

Supplementary Table S3. Treatment modalities and outcomes in 35 patients with GOGNs

Variable	Cases (n)	Percentage
**Treatment modality**		
Endoscopic submucosal dissection (ESD)	14	40%
Surgical resection	2	6%
Cold snare polypectomy	11	31%
Biopsy only / conservative observation	8	23%
**Curability classification after ESD ***		
eCura A (pT1a)	3	21%
eCura B (pT1b)	9	65%
eCura C-1	0	0
eCura C-2	2	14%
**Follow-up outcome ※**		
Alive	35	100%
Disease-specific death	0	0

Table footnotes: * Percentages for curability classification were calculated based on the total number of patients who underwent ESD (n = 14). ※Median total follow-up duration was 27 months (range: 13–70 months). Median endoscopic follow-up duration was 21 months (range: 7–64 months). Abbreviations: GOGNs – gastric oxyntic gland neoplasms

Supplementary Table S4. eCura classification of curative potential applied for gastric oxyntic gland neoplasms (GOGNs) resections.

Classification	Criteria
eCura A (absolute curative)	En bloc resection; differentiated-type dominant histology; pT1a mucosal cancer without ulceration; or tumor < 3 cm, differentiated, pT1a with ulceration; or tumor < 2 cm, undifferentiated, pT1a, no vascular invasion, and negative lateral/vertical margins.
eCura B (expanded curative)	En bloc resection; tumor < 3 cm, differentiated; pT1b submucosal invasion < 500 µm; no vascular invasion; and negative resection margins.
eCura C1 (relative non-curative)	Non-curative resection solely due to piecemeal resection or horizontal margin positivity.
eCura C2 (absolute non-curative)	Failing to meet criteria for A/B/C1. Surgical resection is recommended unless contraindicated or declined by the patient.

Supplementary Figure S1. Representative histopathological and immunohistochemical features of three GOGNs.
 Panels A–F (Case 1) : (A–C) Histopathology shows irregularly arranged fundic gland–type glands with superficial foveolar differentiation, mild cytologic atypia, and focal submucosal invasion. (D–F) IHC: diffuse pepsinogen I (D), scattered strong H^+/K^+ ATPase (E), and extensive MUC6 (F) cytoplasmic positivity.
 Panels G–L (Case 2): (G–I) Histopathology shows compact branching fundic gland–type glands with chief cell–predominant morphology, superficial foveolar differentiation, and focal penetration of the muscularis mucosae. (J–L) IHC: Ki 67 positivity mainly superficial (5%) (J); strong diffuse MUC5AC in superficial epithelium (K); diffuse MUC6 expression in deep glands (L).
 Panels M–R (Case 3): (M–O) Histopathology shows elongated compact fundic gland–type glands with mild-to-moderate atypia and focal disruption of the muscularis mucosae. (P–R) IHC: Ki 67 nuclear positivity (10%) (P); strong diffuse MUC5AC (Q); focal p53 nuclear staining (20%) (R).
 Each case occupies two rows (A–F, G–L, M–R). For each case, a corresponding endoscopic image of the lesion has been inserted as a thumbnail (inset) in the bottom-left corner of the first histology panel of that case (A, G, M). Insets correspond to the lesions shown in Figure 8A–D (Case 1), Figure 4E–H (Case 2), and Figure 4A–D (Case 3).
 All panels: Hematoxylin and Eosin (H&E) or immunohistochemistry (IHC), hematoxylin counterstaining.

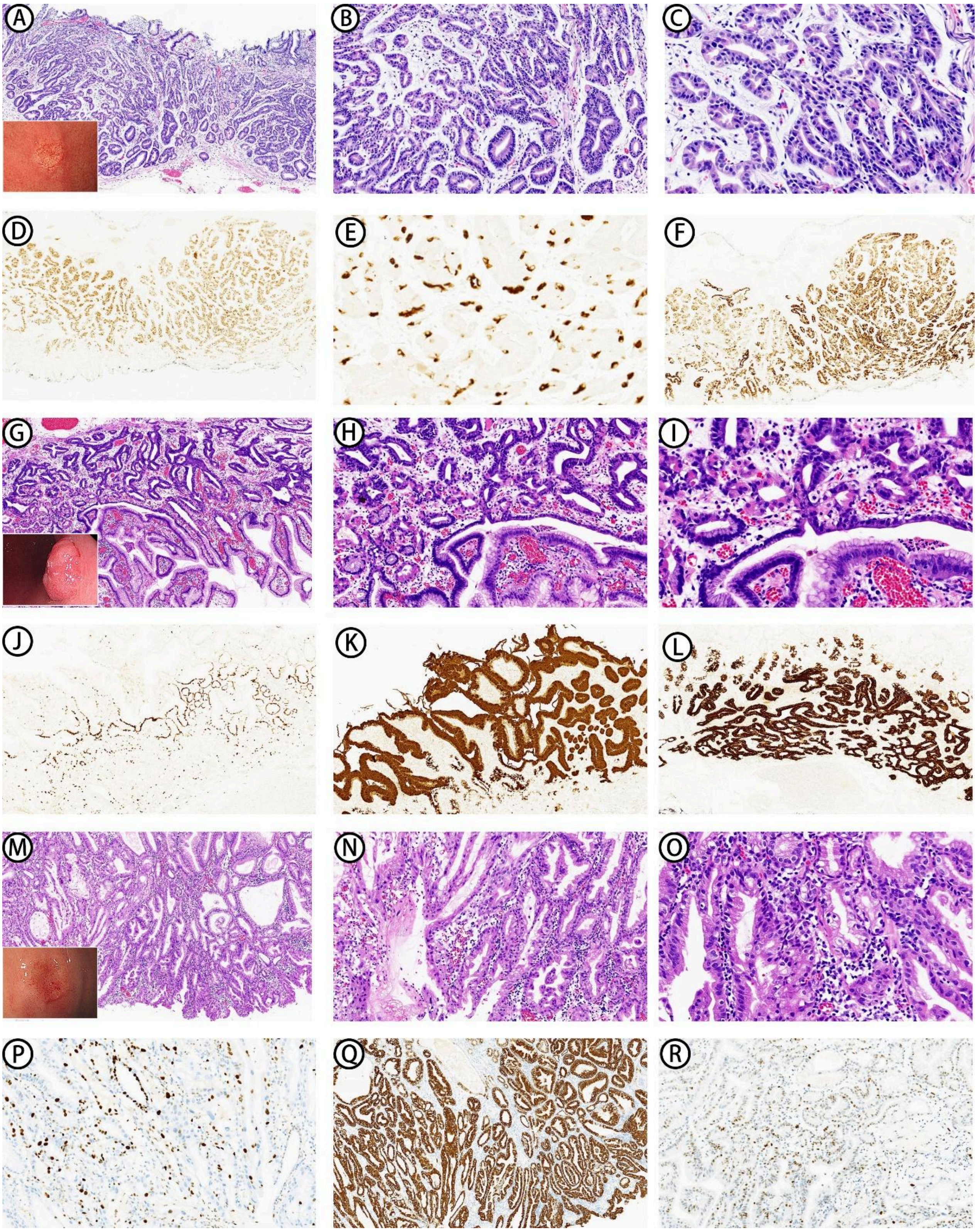
A
B
C
D
E
F
G
H
I
J
K
L
M
N
O
P
Q
R

Supplementary Figure S2. Representative endoscopic, resection, and histopathological findings of two GOGNs treated by ESD.Panels A–D (Case 1): (A) Pre-ESD endoscopy shows a type 0–IIa with a subtly yellowish surface and visible branch-like vessels, located in the upper gastric body. (B) Post-ESD endoscopic view. (C) Macroscopic appearance of the resected specimen. (D) Post-ESD histopathology shows irregular fundic gland–type glands with mild atypia and superficial foveolar differentiation, with focal submucosal invasion (50 μm), consistent with gastric adenocarcinoma of fundic gland mucosa type (GA-FGM).
 Panels A–D (Case 2): (E) Pre-ESD endoscopy shows a type 0–IIb with a yellowish surface and visible branch-like vessels, located in the upper gastric body. (F) Post-ESD endoscopic view. (G) Macroscopic appearance of the resected specimen. (H) Post-ESD histopathology shows gastric oxyntic gland adenoma (OGA), no evidence of submucosal invasion, surrounding gastric mucosa shows chronic atrophic gastritis with moderate inflammation, moderate atrophy, and moderate intestinal metaplasia.
 All pathological panels: Hematoxylin and Eosin (H&E) staining with hematoxylin counterstaining.

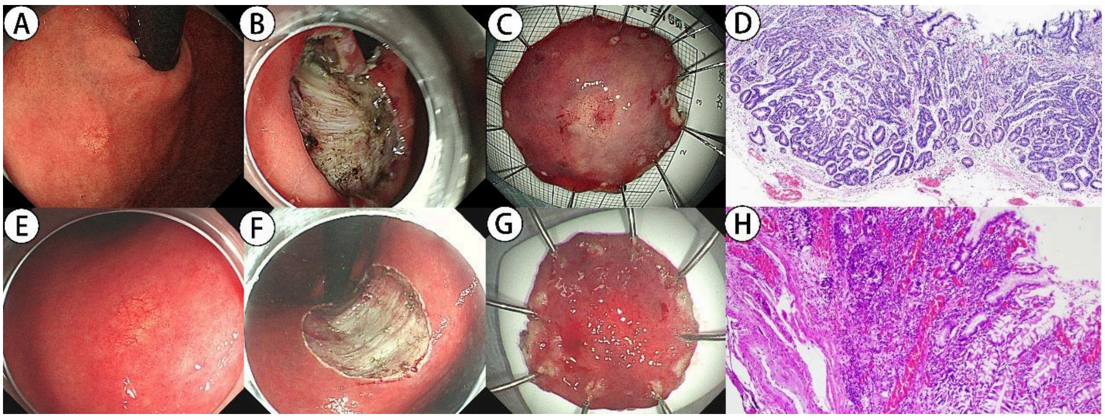

Supplementary Figure S3. Representative endoscopic, resection, and histopathological findings of two additional GOGNs treated by ESD.
 Panels A–D (Case 1): (A) Preoperative endoscopy shows a type 0–IIa+IIc lesion with a subtly reddish surface in the upper gastric body. (B) Post-ESD endoscopic view. (C) Macroscopic appearance of the resected specimen (D) Post ESD histopathology demonstrates GA-FGM with submucosal invasion (1000 µm).
 Panels E–H (Case 2):
 (E) Preoperative endoscopy shows a type 0–IIa lesion with a reddish surface. (F) Post-ESD endoscopic view. (G) Macroscopic appearance of the resected specimen. (H) Post-ESD histopathology shows GA-FGM with focal submucosal invasion (500 µm).
 All pathological panels: Hematoxylin and Eosin (H&E) staining with hematoxylin counterstaining.

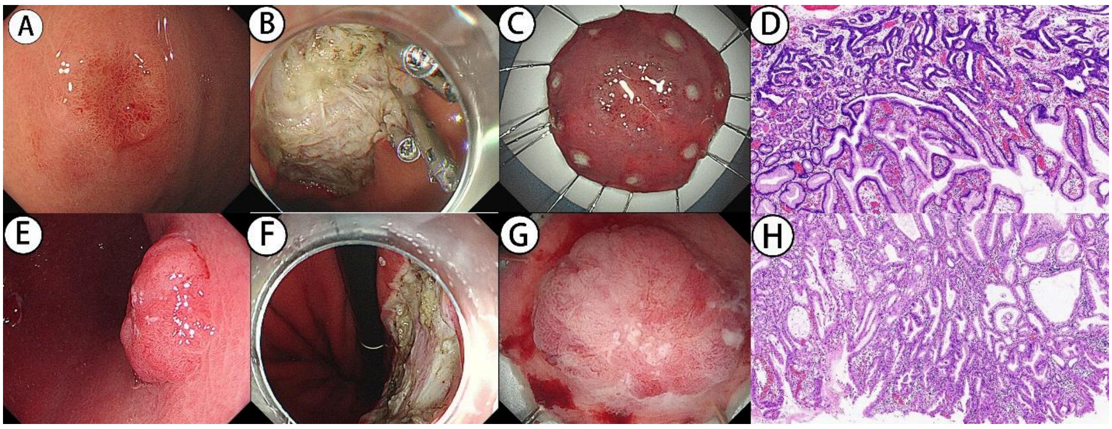

Supplement: Supplementary file 1 — Ergänzendes Material [file 10-1055-a-2889-6638_28930734.pdf]
